# Spatial distribution of supratentorial diffuse gliomas: A retrospective study of 990 cases

**DOI:** 10.3389/fonc.2023.1098328

**Published:** 2023-01-24

**Authors:** Gen Li, Chuandong Yin, Chuanhao Zhang, Bowen Xue, Zuocheng Yang, Zhenye Li, Yuesong Pan, Zonggang Hou, Shuyu Hao, Lanbing Yu, Nan Ji, Zhixian Gao, Zhenghai Deng, Jian Xie

**Affiliations:** ^1^ Department of Neurosurgery, Beijing Tiantan Hospital, Capital Medical University, Beijing, China; ^2^ China National Clinical Research Center for Neurological Diseases, Beijing, China; ^3^ Beijing Advanced Innovation Center for Big Data-Based Precision Medicine, Beihang University, Beijing, China

**Keywords:** supratentorial, diffuse gliomas, spatial disproportion, age, WHO grade, molecular status

## Abstract

**Background:**

Gliomas distribute unevenly in the supratentorial brain space. Many factors were linked to tumor locations. This study aims to describe a more detailed distributing pattern of these tumors with age and pathological factors concerned.

**Methods:**

A consecutive series of 990 adult patients with newly-diagnosed supratentorial diffuse gliomas who underwent resection in Beijing Tiantan Hospital between January 2013 and January 2017 were retrospectively reviewed. For each patient, the anatomic locations were identified by the preoperative MRI, and the pathological subtypes were reviewed for histological grade and molecular status (if any) from his medical record. The MNI template was manually segmented to measure each anatomic location’s volume, and its invaded ratio was then adjusted by the volume to calculate the frequency density. Factors of age and pathological subtypes were also compared among locations.

**Results:**

The insulae, hippocampi, and corpus callosum were locations of the densest frequencies. The frequency density decreased from the anterior to posterior (frontal - motor region - sensory region - parietal - occipital), while the grade (p < 0.0001) and the proportion of IDH-wt (p < 0.0001) increased. More tumors invading the right basal ganglion were MGMT-mt (p = 0.0007), and more of those invading the left frontal were TERT-wt (p = 0.0256). Age varied among locations and pathological subtypes.

**Conclusions:**

This study demonstrated more detailed spatial disproportions of supratentorial gliomas. There are potential interactions among age, pathological subtypes, and tumor locations.

## Introduction

Adult diffuse gliomas are primary central nervous system cancers of a most lethal kind, mainly distributed in supratentorial brain areas (1, 2). Lack of clarity regarding the origins and developing processes contributed mainly to the dissatisfactory treatment outcomes (3–8). Evidence has suggested likely linkages between tumor location and the cellular origin (9, 10). A promising theory suggests that those tumors originate from neural stem cells (NSC) in the subventricular zone (SVZ) and migrate to populate their destinations (11–13). But what determined their destinations remained vague. Meanwhile, there is also evidence suggesting that NSC-derived progeny might also be a potential origin of these tumors (14). Several studies demonstrated the disproportionate distribution of gliomas to a certain extent (15, 16), which should be eventually explained by the theory of oncogenesis. Larger scale studies with fine localization of the lesions are still in need, to reveal a clear distributing pattern, or rather what kinds of spatial nonuniformities are there.

Here, we used a representative case series to describe a more detailed distributing pattern of supratentorial gliomas, with the effects of locations’ volume, pathological subtypes, and age concerned.

## Material and methods

### Patients

**Figure 1:**
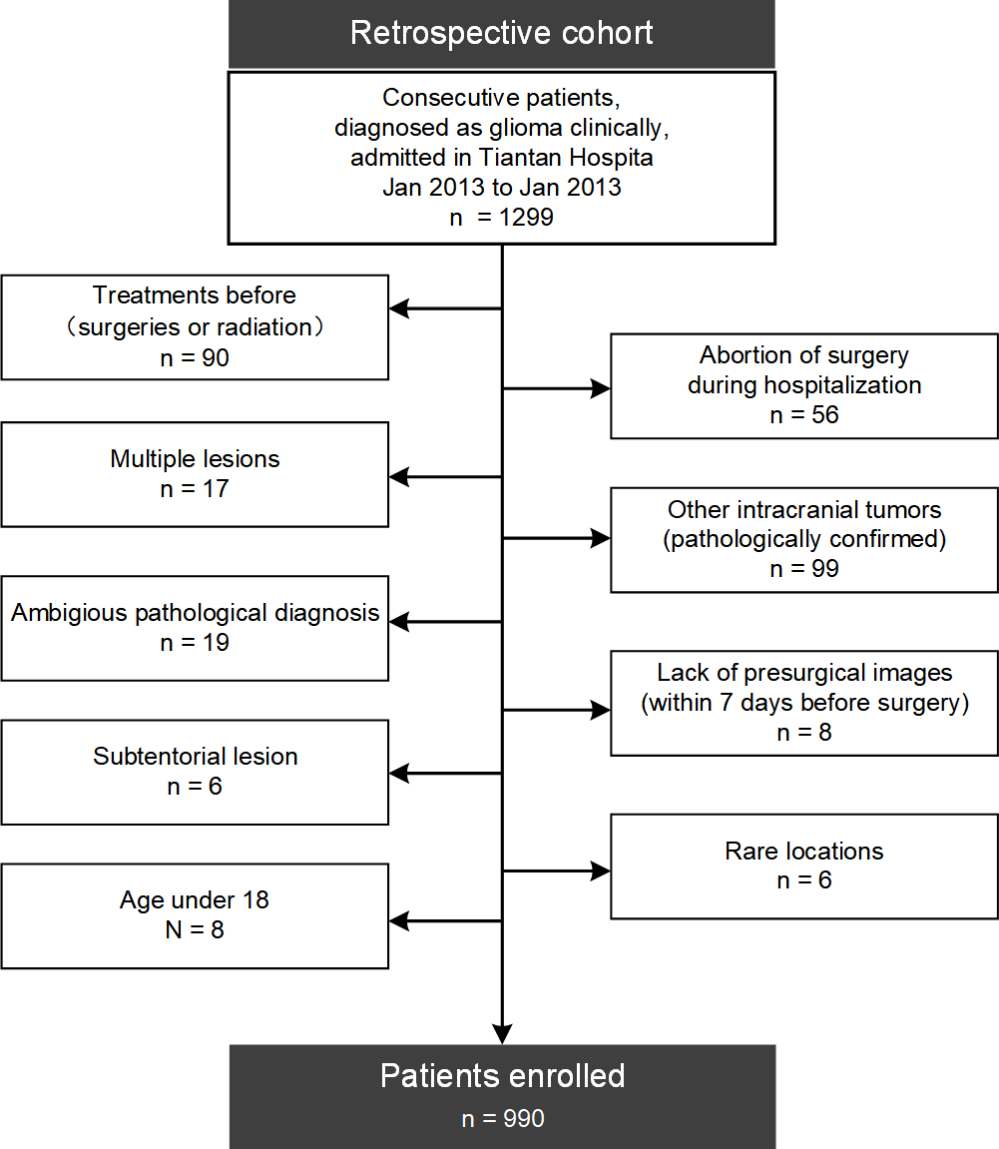
Flow diagram of patients’ enrollment. 990 adult patients with newly-diagnosed supratentorial diffuse gliomas were finally enrolled from a retrospective cohort of 1299 patients in this study.

As shown in the flow diagram of **Figure 1**, patients were screened from a retrospective cohort of 1299 continuous patients with suspected gliomas admitted into Beijing Tiantan Hospital between January 2013 and January 2017, according to the following criteria: (a) without surgical treatment before; (b) single lesion; (c) pathologically confirmed diffuse gliomas; (d) supratentorial locations and not confined within rare locations such as the optic pathway; (e) MRI within 7 days before surgery; (f) adult patients (age ≥ 18).

This study was approved by Beijing Tiantan Hospital institutional review board. Written informed consent was waived for the retrospective nature.

### Anatomic division and the volume

To describe the locations of lesions, we divided firstly, based on the international classification of diseases (ICD-11, https://www.who.int/standards/classifications/classification-of-diseases), the supratentorial brain matter into eight anatomic sites of the “lobular” level (referred to as the Regions), including the frontal, the temporal, the parietal, the occipital and the insulae, as well as those nuclei or white matter areas of the basal ganglia, the corpus callosum, and the thalami, which could not be assigned to the spectral of any single lobe. Besides, four eloquent areas of clear margins (the pre- and postcentral gyrus, the gyrus cinguli, and the hippocampus, referred to as the Subregions) were also considered. Each Region or Subregion was also divided into the left and right ones (or the half for the corpus callosum), taking the anatomic midline of each axial slice as a reference.

An original atlas was manually segmented into those anatomic sites based on MNI-152 (17), the widely recognized standard brain template, in *3D-slicer 4.13.0* (www.slicer.org). The Desikan-Killiany Atlas (18) was taken as a reference for the division of the cortical areas. The white matter was segmented and adjusted in every slice of the axial, sagittal, and coronal views. The volume of each anatomic site was then measured based on this atlas.

### Localization of tumors

Two neurosurgeons (> 10 years of experience, ZH and ZL) reviewed the MRIs of each patient separately to specify the tumors’ locations by anatomic sites. The senior neurosurgeon (>30 years of experience, JX) made the final decision when there were disagreements. For lesions without evident enhancement, the margins were determined by axial T2/FLAIR images. Meanwhile, post-contrast T1 images (in sagittal and coronal views) were also referred to. And for lesions with evident enhancement, post-contrast T1 images in the three views determined the margins together. A lesion can be defined as invading more than one site: taking the natural anatomic margins as references, topologic areas outside the core of the bulk were identified as affected only when the scopes of lesions invaded across those borders exactly. Otherwise, they were not affected when the anatomic margins shifted due to the mass effect without clear lesional signs within the spectra of target sites. For gliomas with Subregions affected, corresponding Regions were also identified as affected.

Furthermore, the number of Regions invaded was also counted (neglecting the lateral, for instance, lesions in bilateral frontal lobes and corpus callosum invaded two Regions: the frontal and the corpus callosum) for each patient. For tumors invading more than one Region (referred to as Multi-regional tumors, and Localized ones for their counterpart), they were regarded as lesions in different locations separately unless otherwise specified. And to evaluate the trends from anterior to posterior, the frontal, motor region, sensory region, parietal, and occipital were indexed as 1 to 5.

### Pathological subtypes

Pathological data were extracted from each patient’s medical records, including histological grades and molecular subtypes (if any) of isocitrate dehydrogenase (IDH) mutation, 1p/19q-codeletion, O(6)-methylguanine DNA methyltransferase (MGMT) methylation, as well as telomerase reverse transcriptase promoter (pTERT) mutation. And for histological grades, lesions were further divided into subtypes of glioblastomas (GBMs, WHO IV) and lower-grade gliomas (LGGs, WHO II or III). Moreover, molecular subtypes were divided as mutant type (-mt) or wildtype (-wt) for IDH and pTERT, co-deleted (-cd) or non-co-deleted (-ncd) for 1p/19q, and methylated (-mt) or unmethylated (-wt) MGMT, respectively. And subtypes of 1p/19q-cd were only considered in those patients of IDH-mt.

### Evaluation of the frequency density

The volume varies widely among locations. Larger areas like the frontal should be more vulnerable even if there were a uniform distribution. Therefore, we calculated the frequency density of each site by adjusting the invaded ratios with its volume. Moreover, to reduce the volume measurement error, it was corrected to two decimal places at the unit of 10^5 mm3. The frequency density was compared among locations to reveal the actual sites of predilection.

### Statistical analysis

All the statistical tests were performed using *Scipy 1.8.0* (https://scipy.org/) in *Python 3.10.2*. The invaded ratios and constitutions of pathological subtypes were compared among sites by chi-square tests, while one-tailed t-tests were used to distinguish the age gaps among sites and between pathological subtypes. Mann-Whitney U test and Spearman’s rank correlation analysis were also applied in this study and are noted otherwise. P < 0.05 was considered statistically significant. The region-based lesion maps illustrating the probability of adult supratentorial diffused gliomas were generated using *Nibabel 4.0.2* (https://nipy.org/packages/nibabel/index.html). And tree-maps of pathological constitutions were drawn using *Plotly 5.6.0* (https://github.com/plotly).

## Results

### Patient demographics

990 adult patients with newly-diagnosed supratentorial diffuse gliomas were finally enrolled in this study. They all had routinely presurgical T2/Flair and post-contrast MRI images acquired within 7 days before the surgery. Histological data were available for every patient, while some had molecular data and others not, as shown in **Table 1**.

**Table 1:** Baseline demographics and pathological characteristics of patients.

Variable	Value
Number of Patients	990
Sex	
Male	593 (59.90%)
Female	397 (40.10%)
Age (years)*	44.24 ± 12.10
Grade	
II	404 (40.81%)
III	240 (24.24%)
IV	346 (34.95%)
IDH	426
Wild-type	113 (26.53%)
Mutant-type	313 (73.47%)
1p/19q	271
Non-codeleted	120 (44.28%)
Codeleted	151 (55.72%)
MGMT	685
Non-methyleted	196 (28.61%)
Methyleted	489 (71.39%)
pTERT	326
Wild-type	154 (47.24%)
Mutant-type	172 (52.76%)

Unless otherwise specified, data are numbers of patients, with or without percentages in parentheses.

*Data are presented as means ± standard deviations.

IDH, isocitrate dehydrogenase; MGMT, O(6)-methylguanine DNA methyltransferase; pTERT, telomerase reverse transcriptase promoter.

Age gaps were there between different pathological subtypes: patients with glioblastoma were older than those with LGGs (p < 0.0001), meanwhile, patients with IDH -wt are older (p < 0.0001), as well as those of pTERT-mt (p < 0.0001). No significant sex gap was found between all the pathological indicators above.

### The susceptibility varies with anatomic sites

For these patients, 531 left hemispheres were affected, while 512 neoplasms invaded the counterpart, among which 53 were bilateral lesions and 56.77% were Localized tumors.

**Figure 2:**
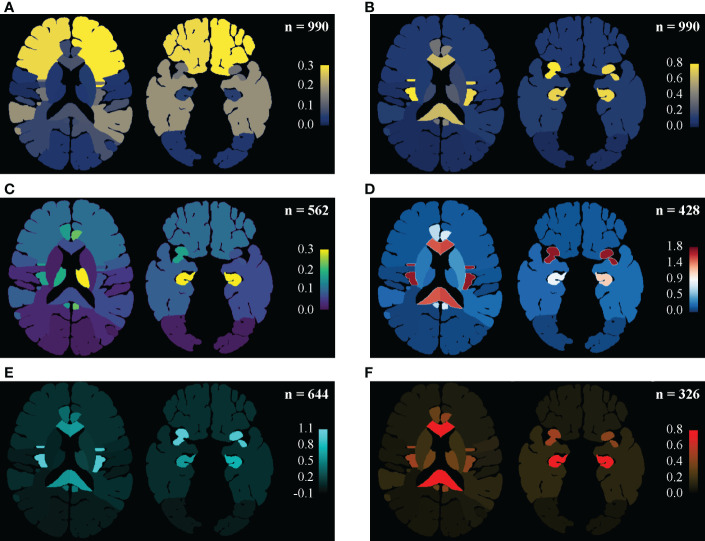
Regine-based lesion map. Distribution pattern of gliomas were illustrated as an entity and in subgroups. **(A, B)** In the entirety of the main set, the distributing pattern is shown in **(A)**, and the frequence density (per 105 mm3) are shown in **(B)**: the frontal and temporal lobes are vulnerable mainly for their remarkable portions of the supratentorial space, while the insulae, hippocampi, corpus callosum and gyri cinguli are real locations of predilection, isolating the effect of volume. Setting the precentral and postcentral gyri as landmarks, decreases in frequency density from the frontal to the occipital was then highlighted. **(C)** Frequency density mapping in sub-groups of the Localized tumors. The thalami (especially the lefr one) stood in this subgroup. **(D)** Frequency density mapping in sub-groups of the Multi-regional tumors. The insulae were highlighted. **(E)** Frequency density mapping in LGGs. Similar disproportion as in the entirety might be due to LGGs predominance. **(F)** Frequency density mapping in GBMs showed that the corpus callosum and hippocampi were more vulnerable to GBMs than the insula.

As shown in **Figure 2** and detailed in **Supplementary Table S1**, the frequencies of invasion were in descending order from the frontal, temporal, and insula to other sites (**Figure 2A**). But, isolating the effect of anatomic volume, the insulae, hippocampi, and corpus callosum became the top 3 locations of predilection (**Figure 2B**), which were stable in almost every sub-groups of Localized (**Figure 2C**) or Multiregional lesions (**Figure 2D**) and LGGs (**Figure 2E**) or GBMs (**Figure 2F**). Taking the motor areas and sensory areas as references, a descending trend of the occurrence dense from the anterior to the posterior (frontal → motor area → sensory area → parietal → occipital) stood out. The temporal, parietal, occipital, insulae, corpus callosum, and basal ganglia were all Regions that tended to harbor more Multi-regional lesions with p-values less than 0.0001, similarly Gyri cinguli (p < 0.0001), and the left hippocampus (p = 0.0136) as Subregions. And Mann-Whitney U tests revealed that among Multi-regional lesions, those with the frontal (p = 0.0020), parietal (p = 0.0287), or occipital (p = 0.0384) in left hemispheres as well as the temporal (p < 0.0001), insulae (p < 0.0001), basal ganglia (p < 0.0001) or thalami (p = 0.0003) in either hemisphere tended to invade more Regions.

### Age gaps among topological locations

Besides those regarding pathological subtypes, age gaps were also found among sites. Patients with affected thalamus in the left (p = 0.0021) or affected frontal (p < 0.0001) were younger, while those with affected temporal (p = 0.0001), parietal (p < 0.0001), or occipital (p = 0.0008) were older. In addition, patients who harbored Localized lesions were younger than those Multi-regional victims (p = 0.0003). Though there were not any significant differences in age found for those with invaded insula, the left (p = 0.0186) and the right (p = 0.0054) affected ones were younger in subgroups of Multi-regional and Localized tumors, respectively. Spearman’s correlation also showed an ascending trend of age from anterior to posterior (**Table 2**, p < 0.0001), but no significant correlation between age and the number of invaded regions (p = 0.8199).

**Table 2:** Anterior-posterior trends in age and pathological constitutions.

Variables		Locations (index)					P_└_
		Frontal	Motor area	Sensory area	Parietal	Occipital	
Number of patients (percentages)		533 (53.84%)	60 (6.06%)	35 (3.54%)	139 (14.04%)	46 (4.65%)	**-**
Age* (n = 990)		42.8555 ± 11.0777	45.5500 ± 11.2120	45.3428 ± 11.0025	49.2302 ± 13.0158	49.7391 ± 13.9259	**< 0.0001**
Grade* (n = 990)		2.7824 ± 0.8166	3.0000 ± 0.8437	3.1143 ± 0.9322	3.3813 ± 0.8111	3.4783 ± 0.7814	**< 0.0001**
IDH (n = 426)	Number of patients (percentage)	240 (56.34%)	27 (6.34%)	18 (4.23%)	62 (14.55%)	18 (4.23%)	**< 0.0001**
	Mutant-type (percentages)	202 (84.17%)	21 (77.78%)	11 (61.11%)	30 (48.39%)	6 (33.33%)	
1p/19q (n = 271)	Number of patients (percentages)	180 (66.42%)	19 (7.01%)	9 (3.32%)	25 (9.23%)	5 (1.85%)	0.4143
	Co-deleted (percentages)	111 (61.67%)	16 (84.21%)	5 (55.56%)	11 (44.00%)	2 (40.00%)	
MGMT (n = 685)	Number of patients (percentages)	356 (51.97%)	44 (6.42%)	25 (3.65%)	104 (15.18%)	34 (4.96%)	0.9796
	Methylated (percentages)	262 (73.60%)	33 (75.00%)	20 (80.00%)	75 (72.12%)	25 (73.53%)	
pTERT (n = 326)	Number of patients (percentages)	180 (55.21%)	19 (5.83%)	14 (4.29%)	48 (14.72%)	16 (4.91%)	0.6612
	Mutant-type (percentages)	89 (49.44%)	11 (57.89%)	5 (35.71%)	20 (41.67%)	13 (81.25%)	

*Data are presented as means ± standard deviations.

**˪**P-value of Spearman’s rank correlation analysis, bold for significant values (p < 0.05).

### The constitution of pathological subtypes varies with sites

**Figure 3:**
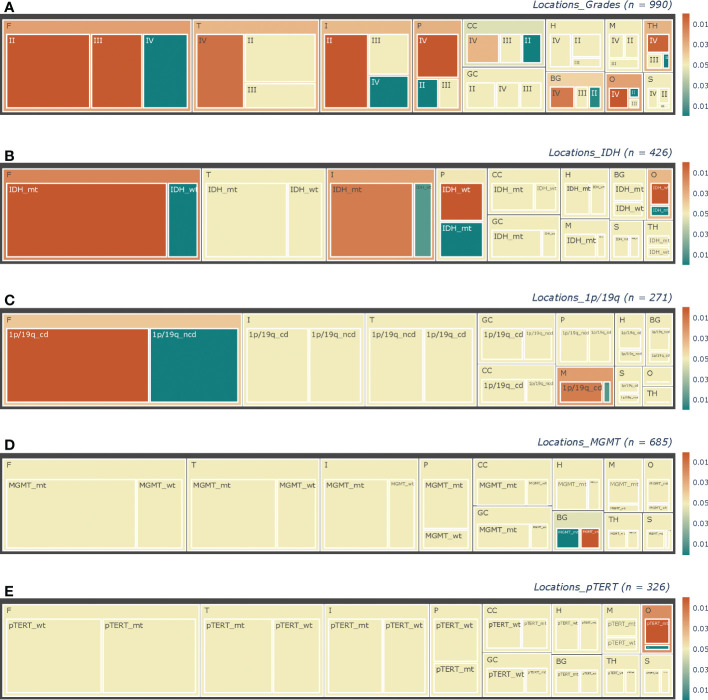
Tree-maps of the pathological constitutions. Constitutions of **(A)** Grade, **(B)** IDH status, **(C)** 1p/19q status, **(D)** MGMT status, and **(E)** pTERT in each anatomic area are respectively shown in diagrams. The lateral factors were not concerned here. Each block represents a subgroup in corresponding hierarchies, with block size showing the ratio of its sample size to the counterpart. The total sample sizes in each chart are noted at the upper right as “(n =)”. As shown in the color bars at the right parts, the color of blocks represented the p-value revealed by chi-square tests, with the yellow color representing those insignificant results of p > = 0.05, while the red and green colors mean “greater” and “less” separately. In addition, the deeper the color is, the smaller the p-value will be. F, Frontal lobes; T, Temporal lobes; P, Parietal lobes; O, Occipital lobes; CC, Corpora Callosa; BG, Basal Ganglia; TH, Thalami; M, Motor regions; S, Sensory regions; H, Hippocampi; GC, Gyri Cinguli; IDH_mt, IDH_mutant type; IDH_wt, IDH_wild type; 1p/19q_cd, 1p/19q_codeleted; 1p/19q_ncd, 1p/19q_nodeleted; MGMT_wt, MGMT_methylated; MGMT_wt, MGMT_wild type; pTERT_mt, pTERT_ mutant type; pTERT_wt, pTERT_ wild type.

Thanks to the large base, the frontal accounted for most in nearly every pathological subtype considered in this study. And as shown in **Figure 3**, pathological factors varied in many aspects. Histologically, for frontal (p < 0.0001) and insulae (p < 0.0001), the proportions of LGGs were larger, which were smaller for the thalamus (p = 0.0009), temporal (p = 0.0141), and basal ganglion (p = 0.0064) in the right, and the parietal (p < 0.0001), occipital (p < 0.0001) lobes (**Figure 3A**). Spearman’s rank correlation analysis revealed an ascending trend of the grade from the anterior to the posterior (**Table 2**, frontal - motor region - sensory region - parietal – occipital, p < 0.0001).

Regarding the molecular status, differences were also there. Firstly, in the frontal (p < 0.0001), IDH-mt lesions were more than IDH-wt tumors, while in the parietal (p < 0.0001) and occipital (p = 0.0002), opposite results were there (**Figure 3B**). And in the insulae, we found a nearly significant p-value of 0.0501 (p = 0.0126, neglecting the factor of lateralization) for the right one, which suggested more lesions were IDH-mt, while insignificant for the left one (p = 0.2856). Furthermore, Spearman’s rank correlation analysis also revealed an ascending trend of the proportion of IDH-wt from the anterior to the posterior (frontal - motor region - sensory region - parietal – occipital, p < 0.0001). Secondly, for 1p/19q, we found nearly significant p-values for tumors invading the frontal (p = 0.0793) or the motor region (p = 0.0624) in the left, which suggested that there might be more 1p/19q-cd tumors and became significant neglecting the factor of lateralization (p = 0.0082 and p = 0.0186 respectively, **Figure 3C**). Next, more of the lesions with the right basal ganglion invaded are MGMT-wt (p = 0.0007), and for GBMs, more of the ones invaded the right temporal were also MGMT-wt (p = 0.0181). No significant differences between MGMT subtypes were found for the left temporal. Finally, left frontal gliomas were more likely to be pTERT-wt (p = 0.0259), while those right frontal (p = 0.0185) and right temporal ones (p = 0.0214) were more of pTERT-mt in LGGs and HGGs respectively.

In addition, none of the pathological subtypes was found to invade significantly more Regions than their counterpart. But concerning these subgroups of Localized lesions only, different from the entirety, the left temporal lobes (p = 0.0180) and the hippocampus (neglecting the lateral factors, p = 0.0058) harbored more HGGs than LGGs.

## Discussion

Developments in genetic and epigenetic research have provided us with pathological indicators as doors into oncogenesis and the progression process of diffused gliomas (19). But, insufficient knowledge of the mechanism deep inside is becoming increasingly challenging in optimizing individual treatments. A state-of-art promising theory of gliomas said that they came from the neurogenic niches in SVZ and migrated alongside neuro-fibers to their destinations then. However, what determined the destinations remained ambiguous. In the meantime, there seems to be little overwhelming evidence ruling out the possibility of non-SVZ populations as a kind of cellular origin (14).

To verify and develop the theory, we should first figure out the distributing pattern. Attention had been paid to it. A pioneering study demonstrated the preference for LGGs in functional regions, including the supplementary motor areas and the insula (15). Another study noticed the differences in volumes of the frontal, temporal, parietal, and occipital and revealed a predilection of the anterior subcortical structures (16). These studies widely expanded our views on the spatial heterogeneity of supratentorial gliomas, with however limited sample sizes and insufficient classification of anatomic locations. Large-sampled studies based on more detailed divisions of anatomic regions are currently indispensable to reveal the exact distribution pattern of the tumors.

In this study, we revealed more of the spatial disproportion based on 990 patients. Firstly, the insulae, hippocampi, corpus callosum are sites of densest occurrence, which were all periventricular regions except the insula. What medicated these lesions “steer by” the adjacent areas of the basal ganglia to populate in a farther region of the insula? Secondly, the previously demonstrated susceptibility of the anterior regions was verified, along with an ascending trend in WHO grade and proportion of IDH-wt to the posterior standing out. May it be that only those more malignant lesions can migrate posteriorly, which was also a minor part of GBMs? Thirdly, lateralization matters in many aspects, and the left hemisphere did not always seem the “dominant” one. Why? Next, although similar in volume, more temporal lesions invaded adjacent regions than were confined within the temporal, which was insignificant for the frontal. Where did the phenomenon derive? These are solid questions for the theory to answer.

Evidence suggests that tumor location has more implications for pathological subtypes than for the treatment and prognosis (10, 20). In this study, the preferences of IDH-mt in the frontal and insulae, as well as those of IDH-wt in the parietal and occipital, were consistent with former reports. But the ascending trend of preference of IDH-wt from anterior to posterior was rarely reported. Frontal gliomas were previously reported more likely to be 1p/19q-cd (20). In this study, we also found nearly significant differences, though there was only partial access to molecular data. Former studies showed in GBMs the lateralization of methylated MGMT to the left temporal and unmethylated MGMT to the right temporal (9, 21). Here, our data also suggested that MGMT-wt were more in the right temporal, but no significant differences in the left temporal compared with other regions. And in our cohort, the right basal also harbored more MGMT-mt than MGMT-wt, which was seldom reported. Differences in race, age, and sex might also contribute in this discrepancy. In addition, a pioneer study suggested that the frontal showed a higher pTERT-mt rate than other regions in LGGs, without distinguishing the laterals (22). Here we further found that the right frontal contributed mainly to the high rate and the left frontal was not significantly different from others. And then, the higher rate of pTERT-mt in the right temporal among GBMs was also seldom reported before.

Volume is an inevitable factor in verifying the hypothesis of distribution. Many atlases segmented the cortical brain zones and deep nuclei (18, 23). But little attention had been paid to the white matter. We found rare reports comprehensively comparing the volumes among lobes and those eloquent areas. It seems impossible to measure individual volumes precisely due to the mass effect of tumors. As a result, this study employed MNI-152, the most widely used standard brain template to represent the crowd. There should be underestimates of the heterogeneity in more than the effect of age and sex, which remains a task for further studies. In addition, the volumes of lesions may also matter. We did not analyze here due to the remarkable sample size. Recent studies employed registration-based methods like voxel-based lesion-symptom mapping analysis to specify the relationship between tumor locations and molecular alterations (9, 10, 24–26). Whereas, those methods may be inherently flawed in distinguishing those shift effects from actual invasion. For instance, a lesion in the motor area with a shifted central gyrus is more likely to be mapped as a tumor in both the motor and the sensory region. These methods might be promising if only the flaws were overcome.

Linkages between age and histo-molecular profile had been noticed (27). Adult and pediatric diffuse gliomas have been identified as 2 distinct kinds(2). But age’s influences on distribution were underestimated. We found those frontal patients were younger, lower in grade, and more of IDH-mt, while those temporal, parietal, or occipital ones were older, higher in grade, and more of IDH-wt. This is consistent with the relationship between age and pathological subtypes: the younger the patients were, the lower the grade and the more IDH-mt would be. Their interactions remained obscure. Which was the initial factor, and which was the ultimate consequence? Or whether there were other initial factors, they were constellations? Future studies are also in need to figure it out.

There were limitations in this study. First, patients were minimally selected to represent the distribution of gliomas better. Hence, we got only partially available molecular data in this study. Incomplete access to molecular data may compromise the efficacy of this study in molecular status, which is a major limitation. Therefore, only the constitutions of molecular subtypes in anatomic locations, rather than the distribution of a molecular subtype, were analyzed. Next, patients were retrospectively enrolled in this study. There might be selection bias at admission. And then the single-center nature is another limitation. Although minimally screened and large-sampled patients were analyzed, the cohort might not identically represent the population of supratentorial diffuse gliomas. Future multicentric and prospective studies are still in need.

In conclusion, our study demonstrated more details of the spatial distribution, and pointed the potential interactions among age, pathological types and anatomic locations. These will be questions to answer before we thoroughly figure the actual mechanism of oncogenesis and progress out.

## Data availability statement

The raw data supporting the conclusions of this article will be made available by the authors, without undue reservation.

## Ethics statement

The studies involving human participants were reviewed and approved by Beijing Tiantan Hospital institutional review board. Written informed consent for participation was not required for this study in accordance with the national legislation and the institutional requirements.

## Author contributions

GL, ZD, and JX conceptualized and designed the study. LY, NJ, ZG and JX provided the cohort data. SH screened eligible patients. CY, CZ, BX, and ZY collected the data. ZH, and ZL reviewed presurgical images and identified the tumor locations. GL and YP analyzed the data. GL segmented the MNI-152 template, measured the volumes, and drafted the manuscript. ZD and JX critically revised the manuscript. All authors contributed to the article and approved the submitted version.
